# Efficiency of colored modified box traps for sampling of tabanids

**DOI:** 10.1051/parasite/2014068

**Published:** 2014-12-17

**Authors:** Stjepan Krčmar, Vanja Radolić, Petar Lajoš, Igor Lukačević

**Affiliations:** 1 Department of Biology, J.J. Strossmayer University of Osijek Cara Hadrijana 8/A HR-31000 Osijek Croatia; 2 Department of Physics, J.J. Strossmayer University of Osijek Trg Lj. Gaja 6 HR-31000 Osijek Croatia

**Keywords:** Diptera, Tabanidae, Box trap, Color

## Abstract

The efficiency of ten differently colored modified box traps for collecting tabanids was studied in the Monjoroš Forest in eastern Croatia. A total of 5,436 specimens belonging to 16 species of tabanids grouped into six genera were collected. The genus *Tabanus* was the most represented with 98% of all collected tabanids. *Tabanus bromius* comprised 90% of tabanids collected, and was the most abundant species collected in all box traps. The majority of tabanids (74%) were collected from black, brown, bordeaux, red, and blue traps (dark group), whereas 26% were collected from green, light violet, white, orange, and yellow traps (light group). The black modified trap was the most successful and collected 20% of all collected tabanids, whereas the yellow trap was the least effective with 1%. The number of collected specimens of species *T. bromius* differed significantly between the dark and light group of traps. Traps with lower reflectance from green color collected 77% of *T. bromius*. The most species of tabanids (12) was collected in the brown trap, whereas the least number of species (6) was collected in the yellow trap.

## Abstract (fr)

L’efficacité pour la collecte de taons de dix boîtes pièges modifiées et de coloration différente a été étudiée dans la forêt de Monjoroš, à l’est de la Croatie. Au total, 5436 individus ont été collectés, appartenant à 16 espèces et six genres. Le genre *Tabanus* était le plus fréquent, avec 98 % des taons collectés. L’espèce *Tabanus bromius* représentait 90 % des collectes et était la plus abondante dans toutes les 10 boîtes pièges. La majorité des taons, 74 %, a été capturée dans les pièges noirs, bruns, bordeaux, rouges et bleus (groupe sombre), tandis que 26 % ont été capturés dans les pièges verts, violets clair, blancs, oranges, et jaunes (groupe clair). Le piège modifié noir a eu le plus de succès avec 20 % des taons capturés, tandis que le jaune a été le moins efficace, avec 1 % des taons capturés. Le nombre de *T. bromius* capturés diffère significativement entre le groupe des pièges sombres et celui des pièges clairs. Les pièges de réflectance de la couleur verte plus faible ont collecté 77 % des *T. bromius*. Le plus grand nombre d’espèces de taons (12) a été collecté dans le piège brun, tandis que le plus petit nombre d’espèces (6) a été collecté dans le piège jaune.

## Introduction

Female tabanids are nuisance pests for people and livestock because of persistent biting behavior and blood ingestion [6]. Host-seeking female tabanids deserve special attention because they may serve as mechanical vectors of some disease agents to human beings and livestock [14]. High populations of tabanids have a significant economic impact on livestock production and outdoor activities [6]. Throughout the world, the effectiveness of several types of traps has been studied for possible use in controlling tabanid populations. Generally, experiments in trapping tabanids with different types of traps have been carried out in North America. During the 1960s and 1970s, colored tent-like modified Malaise traps were found to be an excellent trap for collection of female tabanids [36, 44]. In studies with several types of Malaise traps in North America, Roberts [36, 37] showed that aside from the design of traps, the trap’s color and the presence of a decoy (black ball) have a significant impact on the quantity of the tabanids collected in the trap. Also, a helio-thermal trap was found to be very effective in trapping tabanids [41]. The helio-thermal trap is based on the greenhouse principle and uses the thermal attraction and the positive phototaxis of adult females [11]. The Manitoba fly trap was presented by Thorsteinson et al. [42]. This trap is rather similar to the helio-thermal trap and consists of a cone-shaped canopy opened at the bottom, which stands on three legs [42]. A large black ball under the cone attracts tabanids into the trap [42]. Several years later, the Manitoba trap was radically redesigned into tent-like canopy traps, from the cone to a four-sided pyramid configuration with a clear top and black bottom [10, 18, 24]. Depending on the design, the Manning fly trap with a brown decoy, and the modified Manning trap, the so-called Horse Pal fly trap, are very similar to the Manitoba trap and both types have been used successfully in tabanid collection [16, 47]. The black, blue, and red colored panels and dark two- or three-dimensional objects have been reported to be an excellent tool for the collection of tabanids [2, 8, 9]. Also, in North America, black, blue, and green box traps have been used successfully to control the abundance of species *Tabanus nigrovittatus* [46]. In studies with the colored NG2G trap in Japan, blue and red colors were effective for all species [39]. Recently in Africa, the Nzi trap was developed for sampling of tsetse and other biting flies; it is a 1 m triangular blue/black cloth trap with two blue wings [31, 32]. The standard cloth Nzi trap or the painted plywood Nzi trap has been very effective in tabanid collection in North America, tropical Australia, and Europe [5, 32, 33, 45]. Traps for tabanid collection are mainly designed to attract tabanids by color, heat, odor, and light polarization [7, 12, 13, 15, 22, 23, 25, 27, 28, 31, 34]. Despite the many field studies throughout North America and Japan about the color attractiveness for tabanids, similar studies in Europe are lacking. The aim of this paper is to present data obtained in field studies and to compare the effectiveness of ten differently colored modified box traps in the collection of tabanids in the European environment.

## Materials and methods

**Figure 1.**
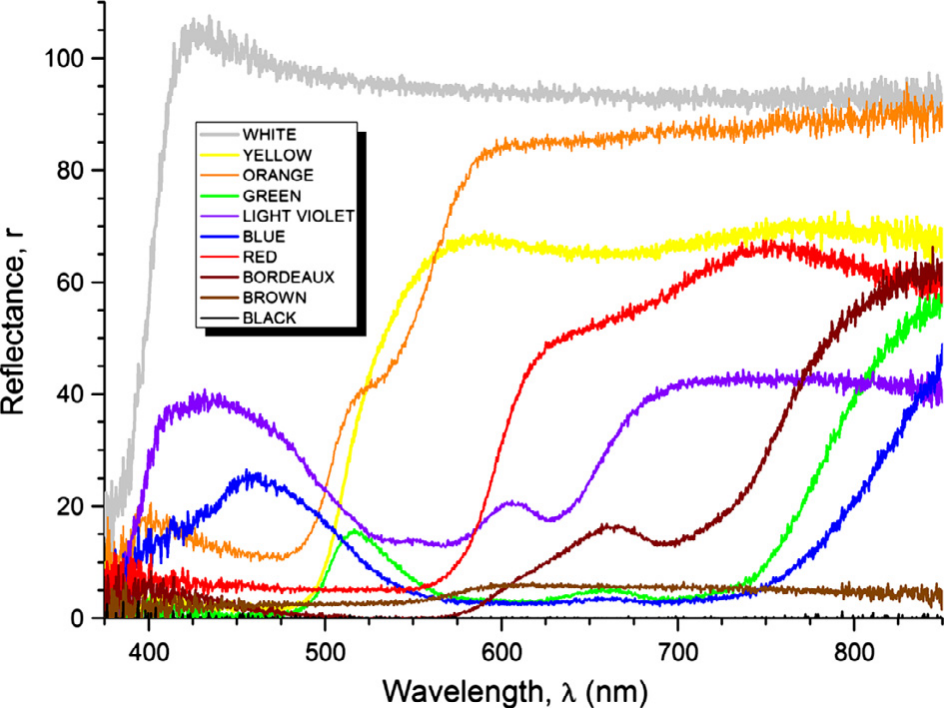
Reflection spectra of the used colors.

**Figure 2.**
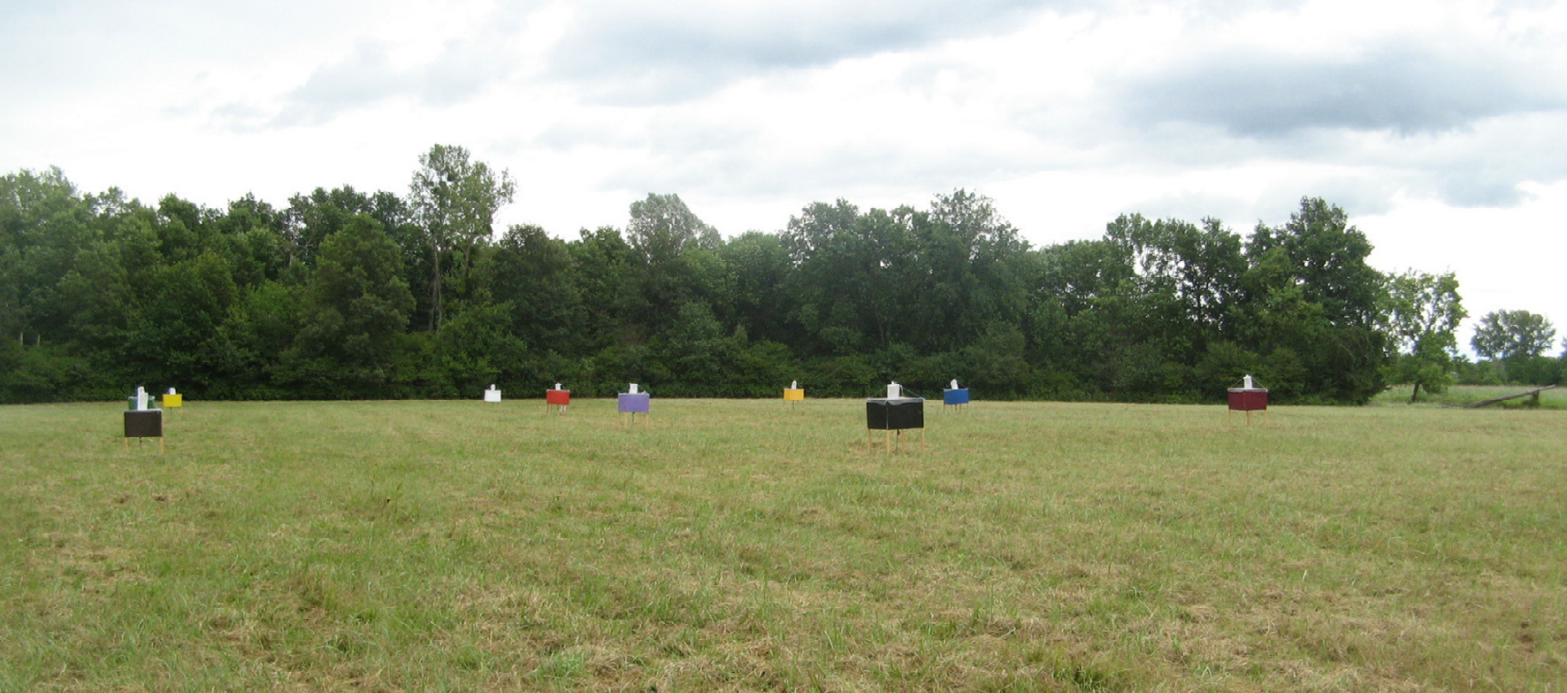
Location and position of modified box traps on the experimental field in Monjoroš Forest (first set of traps: 1 – black, 2 – brown, 3 – bordeaux, 4 – light violet, 5 – green, 6 – blue, 7 – red, 8 – yellow, 9 – orange, 10 – white; the second set was constructed by mirror symmetry along a line connecting traps 5 and 6).

The study was carried out in the Monjoroš Forest (UTM CR 37), (45° 45′ N, 18° 52′ E) in eastern Croatia. The Monjoroš Forest is located along the west bank of the Danube River and is mostly composed of white willow, black poplar, and common oak. The largest part of the Monjoroš Forest lies between 78 and 82.5 m above sea level. Tabanids were sampled on an experimental field by ten homemade colored modified box traps constructed according to the design [19]. The modified box traps consisted of an 80 × 60 cm four-sided plywood box set at 80 cm above ground, attached to the four laths, open underneath, and with a metal insect net on the top. Instead of two diagonally positioned collecting cones, the modified box trap has a 20 cm wide opening in the center of the insect net, with a removable collecting cap made of polyester mosquito mesh positioned on the opening. The outside of each box trap was painted in different colors: brown, black, bordeaux, light violet, green, red, blue, yellow, orange, and white; the inner sides were left unpainted. Painting was done with acrylic colors (Genius Pro, J.W. Ostendorf GmbH & Co. KG, Coasfeid, Germany). Quantitative assessment of colors was examined by using fiber optics UV-VIS reflection spectroscopy (UV-VIS FORS). For the reflection spectroscopy, we utilized a USB2000 UV-VIS spectrometer from Ocean Optics with a detector range of 200–850 nm, optical resolution of about 1.5 nm, silicon CCD array detector, and 600 lines per millimeter grating. The external light source was a tungsten halogen light bulb of spectral range 360–2000 nm. For signal acquisition, we used a fiber optic probe (optimized for UV-VIS range), with a reflection probe holder, in a diffuse mode of reflectance at an angle of 45° with respect to the board surface, as the surface of boards was rough and dull. This mode of operation allowed us to gather a significant amount of reflected signals. Reflection spectra were recorded with respect to the diffuse reflectance standard, which reflects more than 95% of light in the range 250–2,000 nm (Fig. 1). The colored traps were placed in the middle of a meadow in three rows 15 m apart, about 30 m from the forest edge. The distance between the traps in the row was 20 m (Fig. 2). This distance between trap rows eliminates the influence of the canopy shadow of trees from the forest edge on the first and third row of traps. The distance between traps in the rows corresponds to those of similar studies carried out in Japan and the USA [20, 39]. During the sampling period, the placement of the traps was changed every 5 days because the handling with a modified plywood box trap and their moving is not easy due to their fragile construction. Instead of hanging glossy black spheres in the modified box trap, octenol was used as an attractant. All ten traps were baited every day with 4 mL of octenol (1-octen-3-ol, 98% pure; Sigma-Aldrich Chemie GmbH, Steinheim, Germany). This attractant was dispensed from plastic vials of 50 mL volume, while the aperture diameter of the plastic vials was 4.5 cm. Vials were attached to wooden poles placed in the center of the modified box traps at 100 cm height. The daily trapping period was between 7 a.m. and 7 p.m. A total of 25 samplings were made in July 2011 and between June and July 2012. The largest number of tabanids 18% was collected on the first day of the study on July 6, 2011, whereas few tabanids 0.12% were collected on July 19 and 20, 2011 due to unfavorable meteorological conditions. During the other days of study, a more or less equal number of tabanids were collected. During the study period, the air temperature and relative humidity were recorded every hour using a data logger (HE 174, Guangdong, China). The average evaporation volume of octenol per day was 0.5 mL (from 0 to 1.9 mL) and the positive correlation between evaporated volume of octenol and daily temperature was observed. The correlation coefficient was *r* = 0.643; positive and statistical significance was shown by the test with Kendall’s variable (*t* = 3.566 > *t*_0_ = 2.101; this is limiting theoretical value of the Student’s variable for the significance level of 0.05 and 18° of freedom). All trapped tabanids were preserved in ethanol. Identification and nomenclature followed that of [11, 29]. The comparison between the numbers of tabanids collected in different colored modified box traps was analyzed by *t*-test, *F*-test, and Fisher’s PLSD test at *p* < 0.05. All statistical analyses were performed by using Statistica 12 software [40].

## Results

**Figure 3.**
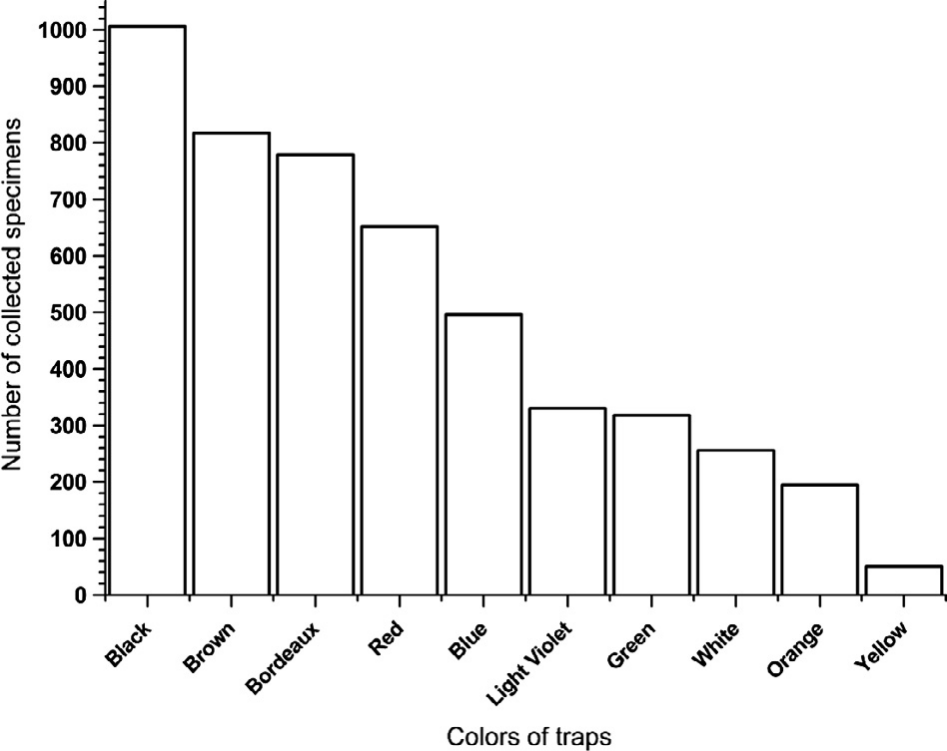
Number of collected specimens of *T. bromius* in different colored traps.

**Figure 4.**
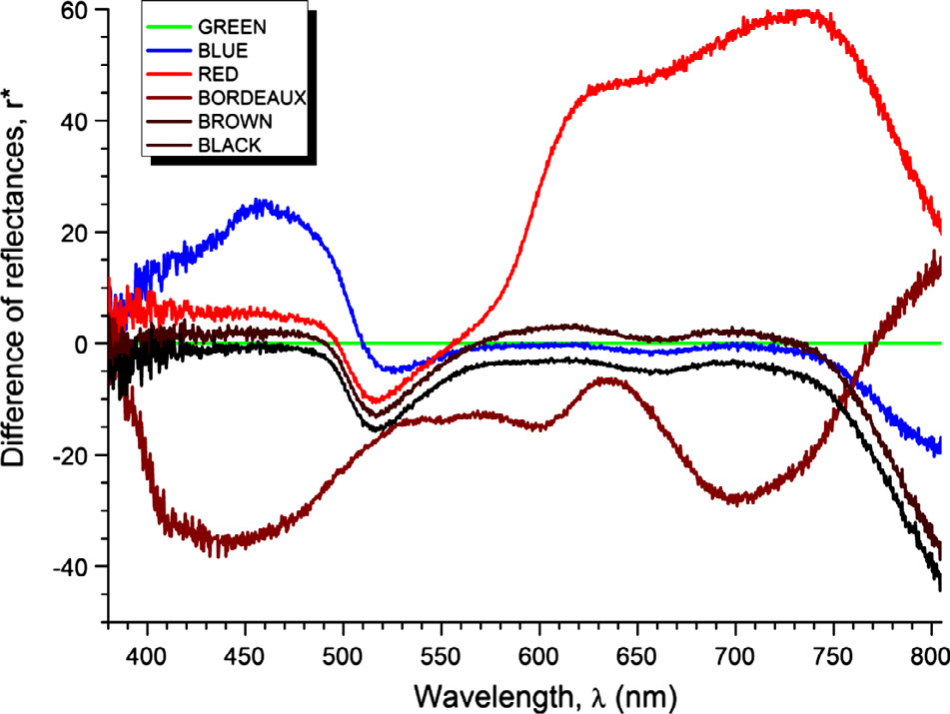
Differences of reflectances of dark-colored traps and green color.

**Figure 5.**
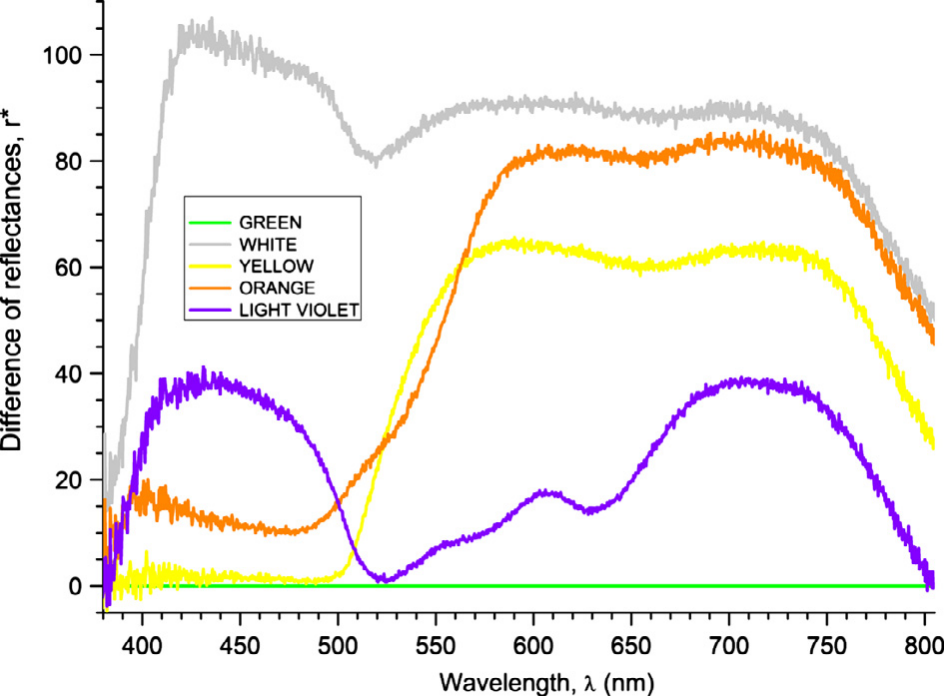
Differences of reflectances of light-colored traps and green color.

A total of 5,436 specimens were collected, belonging to 16 species of tabanids grouped into the following genera: *Silvius*, *Chrysops*, *Atylotus*, *Hybomitra*, *Tabanus*, and *Haematopota* (Table 1). *Tabanus* was the best represented genus with six species and 5,347 specimens, followed by the genera *Atylotus* and *Haematopota* with three species and 45 and 38 specimens, respectively, *Hybomitra* with two species and four specimens, *Silvius* and *Chrysops* with one species and one specimen each (Table 1). A total of 98% of tabanids collected belong to the *Tabanus* genus. The most commonly collected species in all ten colored traps was *Tabanus bromius* L.; this species comprised 90% of the tabanids collected. *Tabanus tergestinus* Egger was the second most abundant species with 5%, while the remaining 14 species made up 5% (Table 1). The black modified box trap collected the most tabanids 20%, followed by the brown one with 17%, the bordeaux with 15%, the red with 13%, the blue with 10%, the green with 7%, the light violet with 7%, the white with 6%, the orange with 5%, and the yellow with 1% (Fig. 3). To sum up, the majority of tabanids (74%) were collected from black, brown, bordeaux, red, and blue traps, whereas 26% were collected from green, light violet, white, orange, and yellow traps. All ten colored modified box traps were classified in two groups, darker colored traps (black, brown, bordeaux, red, blue) and lighter colored traps (green, light violet, white, orange, and yellow). The *t*-test analyses of the trapping data showed significant differences in the number of collected tabanids between the darker and lighter group of traps (*t* = 4.719, *p* < 0.001, *df* = 8). The influence of color on the number of collected specimens was analyzed only for *T. bromius*, because 90% of all collected specimens belong to this species and these numbers of specimens collected from the darker and the lighter groups of colored traps differed significantly (*t* = 5.241, *p* < 0.001, *df* = 8). This result was expected because 34% of specimens of *T. bromius* were collected in black and brown traps. The response of the other 15 species to colored traps was not analyzed because of small sample sizes. The *t*-test revealed that there was no significant site effect on the number of collected tabanids with ten differently colored traps for all collected species and especially for *T. bromius* (all probabilities of *t*-distribution are higher than 0.05). Four dark-colored traps, the black, brown, bordeaux, and blue one showed a lower value of reflectance from green color, while for red it is true only for wavelengths between 500 and 550 nm (Fig. 4). However, the red (except in the range of 500–550 nm) and all light-colored traps showed a higher value of reflectance from green color (Figs. 4 and 5). This study showed that traps with lower reflectance from green color collected 77% of *T. bromius*. In both groups of traps, 13 species of tabanids were collected. The most species of tabanids (12) were collected in the brown modified box trap, followed by black traps with 9 species, whereas 8 species were collected in bordeaux, red, blue, green, light violet, and orange traps (Table 1). The least number of species (6) was collected in the yellow box trap (Table 1).

**Table 1.** List of total number of tabanids collected by ten colored modified box traps.

Species/box trap	Black	Brown	Bordeaux	Red	Blue	Green	Light violet	White	Orange	Yellow
*Silvius alpinus* (Scopoli, 1763)	–	–	–	–	–	–	–	1	–	–
*Chrysops relictus* (Meigen, 1820)	–	1	–	–	–	–	–	–	–	–
*Atylotus flavoguttatus* (Szilády, 1915)	2	–	–	–	1	2	1	–	–	–
*A. loewianus* (Villeneuve, 1920)	9	7	5	4	3	1	4	–	4	1
*A. rusticus* (L., 1767)	–	1	–	–	–	–	–	–	–	–
*Hybomitra ciureai* (Séguy, 1937)	1	1	–	–	–	–	–	1	–	–
*H. ukrainica* (Olsufjev, 1952)	–	1	–	–	–	–	–	–	–	–
*Tabanus autumnalis* L., 1761	15	14	11	7	1	23	1	6	5	1
*T. bovinus* L., 1758	–	7	2	2	–	–	1	–	–	–
*T. bromius* L., 1758	1,006	817	779	652	496	318	330	256	195	50
*T. maculicornis* (Zetterstedt, 1842)	7	5	6	6	4	1	1	2	4	–
*T. sudeticus* (Zeller, 1842)	7	7	5	2	1	11	5	2	4	1
*T. tergestinus* (Egger, 1859)	18	43	21	23	16	37	40	34	35	4
*Haematopota crassicornis* (Wahlberg, 1848)	–	–	–	–	–	–	1	–	–	–
*H. pluvialis* (L., 1758)	8	3	3	1	3	4	3	5	5	1
*H. subcylindrica* (Pandellé, 1883)	–	–	–	–	–	–	–	–	1	–
Σ 16	1,073	907	832	697	525	397	387	307	253	58

## Discussion

The present study showed that black, brown, bordeaux, blue, and red box traps were highly attractive to tabanids, significantly more than green, light violet, white, orange, and yellow traps. In North America, Bracken et al. [8] found that dark colors such as black, blue, and red were highly attractive for tabanids. Also, in studies with colored silhouettes it was found that the object differs from the background in either color or reflectance to be perceived by tabanids [8, 36]. In our study, the most abundant species was *T. bromius*, which comprised 90% of the tabanids collected. The number of specimens of *T. bromius* collected from the darker and the lighter groups of colored traps differed significantly. The black-colored modified box trap was the most attractive to *T. bromius*, the brown-colored trap was the second. However, there is no significant difference between the catches of black and brown colored traps. A similar result was obtained in Japan where black and blue traps collected significantly more specimens of *Hirosia iyoensis* than other colored traps, but without a significant difference between them. Also, in the same study, significantly more specimens of *Tabanus nipponicus* were collected in red traps than in other colored traps. In the USA, Moore et al. [35] found that there was no difference in numbers of *Tabanus abactor* collected between different trap designs, whereas the color significantly affected the number of collected specimens [35]. The red, brown, and black colored traps collected significantly more specimens of *T. abactor* than any other color [35]. In both studies, in Japan and the USA, it was found that yellow traps were less attractive for *Hi. iyoensis*, *T. nipponicus*, and *T. abactor* [35, 39]. Identical results were obtained in our study, in which the yellow-colored trap was less attractive for *T. bromius*, while the red and bordeaux modified box traps collected 29.21% of *T. bromius*. The red-absorbing visual pigment has never been recorded in tabanids, and is not considered to be present [39], which is the reason why red would have appeared as dark gray or black to tabanids. Because of this, a great number of specimens of *T. bromius* were collected in red or bordeaux modified box traps. Allan and Stoffolano [2] reported that *Tabanus nigrovittatus* preferred blue, black, and red panel traps. In our study too, blue, black, and red colored traps collected 44% of specimens of *T. bromius*. Furthermore, Mihok [31] reported that in Kenya blue/black-colored Nzi traps attracted many genera of the family Tabanidae, including species that had never been caught in traps. Color contrast is important in visual attraction of tabanids to traps [1, 3, 39]. Dark blue color showed maximum contrast to the background of grass [39]. In some experiments throughout the world, black, brown, blue, and red traps showed similar results as the ones recorded in our study (77% of *T. bromius* were collected in darker-colored traps), which is mainly based on the reaction of tabanids to dark colors. Blue and green photoreceptors have been detected in *T. bromius* with a peak at 480 nm and 515 nm [30]. This is probably the reason why *T. bromius* was collected at a very high percent in dark-colored box traps. Some North American tabanids are photopositive to light between 375 and 430 nm and between 500 and 550 nm [17]. However, in the North American tabanid fauna, spectral sensitivity of the eye is known only for the species *T. nigrovittatus* Macquart; the eye of this species has high sensitivity in the blue-green region [4]. Furthermore, Browne and Bennett [9] reported that North American species from the genera *Chrysops* and *Hybomitra* preferred blue and red silhouettes, but were not attracted to yellow, black and white silhouettes. Opposite results were recorded in our study because none of the species from the genera *Chrysops* and *Hybomitra* were collected in blue or red traps. Bracken et al. [8] found that *Hybomitra illota* was attracted nearly equally to black, gray, and white silhouettes. This is similar to our results for *Hybomitra ciureai*. However, *H. ciureai* was collected in small numbers, although 75% of the specimens from the *Hybomitra* genus belong to this species. In the middle of the last century, Thorsteinson et al. [42, 43] showed that adult tabanids were strongly attracted visually to a glossy black sphere suspended in the trap, probably due to a highlight or a beam of polarized light. This finding was recently developed by Horvath’s working group, which resulted in the discovery of positive polarotaxis in tabanids [22]. Horizontally polarized light attracts water-seeking males and females [21, 22, 26], while linearly polarized light attracts host-seeking females [12]. This is the case for many traditional traps [21]. However, polarization sensitivity has not been studied adequately at the structural level in the eyes of tabanids and may differ among species [6]. Also, spectral sensitivity may vary with its physiological state [38]. Further studies would contribute to better understanding of the relative importance of visual cues in the behavior of tabanids. This may imply the use of physiological and behavioral techniques.
